# Surgical spacer placement prior carbon ion radiotherapy (CIRT): an effective feasible strategy to improve the treatment for sacral chordoma

**DOI:** 10.1186/s12957-016-0966-6

**Published:** 2016-08-09

**Authors:** Lorenzo Cobianchi, Andrea Peloso, Barbara Vischioni, Denis Panizza, Maria Rosaria Fiore, Piero Fossati, Viviana Vitolo, Alberto Iannalfi, Mario Ciocca, Silvia Brugnatelli, Tommaso Dominioni, Dario Bugada, Marcello Maestri, Mario Alessiani, Francesca Valvo, Roberto Orecchia, Paolo Dionigi

**Affiliations:** 1 Department of Clinical, Surgical, Diagnostic and Paediatric Sciences, University of Pavia, Pavia, Italy; 2 IRCCS Policlinico San Matteo Foundation, General Surgery 1, Pavia, Italy; 3 Department of Radiation Oncology and Medical Physics, Centro Nazionale Adroterapia Oncologica (CNAO), Pavia, Italy; 4 Department of Radiation Oncology, European Institute of Oncology (IEO), Milan, Italy; 5 IRCCS Policlinico San Matteo Foundation, Department of Onco-Hematology, Oncology Section, Pavia, Italy; 6 Department of Surgical Science, University of Parma, Parma, Italy; 7 Department of General Surgery, IRCCS San Matteo Foundation, University of Pavia, Piazzale Golgi, Pavia, 27100 Italy

**Keywords:** Chordoma, Carbon ion radiotherapy, Spacer placement

## Abstract

**Background:**

Sacral chordoma (SC) is a neoplasm arising from residual notochordal cells degeneration. SC is difficult to manage mainly because of anatomic location and tendency to extensive spread. Carbon ion radiotherapy (CIRT) is highly precise to selectively deliver high biological effective dose to the tumor target sparing the anatomical structure on its path even if when SC is contiguous to the intestine, and a surgical spacer might be an advantageous tool to create a distance around the target volume allowing radical curative dose delivery with a safe dose gradient to the surrounding organs. This paper describes a double approach—open and hand-assisted laparoscopic—for a silicon spacer placement in patients affected by sacral chordoma undergoing carbon ion radiotherapy.

**Methods:**

Six consecutive patients have been enrolled for surgical spacer placement—open (three) or hand-assisted (three)—prior carbon ion radiotherapy treatment in order to increase efficacy of carbon ion radiotherapy minimizing its side effects.

**Results:**

Results showed that silicon spacer placement for SC treatment is feasible both via laparoscopic and laparotomic approach.

**Conclusions:**

Its use might improve CIRT safety and thus efficacy for SC treatment.

## Background

Worldwide, with an amount of 1–4 % of all malignant bone tumors, sacral chordoma (SC) is a rare neoplasm belonging to the family of chordomas, a type of tumor arising through malignant transformation of benignant residual notochordal cells that could hypothetically arise from all the length way of the skeletal neuraxis [1]. SC represents around 30 % of all chordomas locations [2] with a commonly slow-growing, chemo/radioresistant, and locally destructive behavior [3]. Hitherto, the achievement of R0 margin after surgical resection is the primary prognostic factor influencing local control. The mortality related to SC is nearly invariable due to their local unrestrainable progression, their common local recurrence (ranging from 43 to 85 % [4]) after surgical resection, and finally, to their very poor sensitivity to any chemotherapy [5] and conventional photon-based radiotherapy (RT) [6].

Carbon ion radiotherapy (CIRT) has recently emerged as very promising strategy for unresectable SC [7]. Charged particles (carbon ions and protons) provide superior dose distribution to non-surface tumors compared with photon RT due to their unique physical properties. In particular, when penetrating the body, their peculiarity compared to photon and proton RT is the release of low radiation dose along their travel path, except for the maximum energy at the end of their range (Bragg peak), with no dose beyond that. This pattern allows to selectively irradiate the tumor target by sparing the surrounding normal tissues. Furthermore, biological experiments in vitro cell line models have shown the higher tumoricidal potential and thus higher biological effectiveness of carbon ion beams compared to the other beam qualities, possibly responsible for the promising results in different SC patient series [8].

The National Center for Oncological Hadrontherapy (CNAO) is the first center in Italy and second in Europe to have started for cancer patient treatment with a dual beam (proton and carbon ions) particle therapy equipment.

High doses are required in the curative RT setting for SC patients. The large and small intestines are very susceptible to radiation dose in general, with surgical adhesions or gastrointestinal bleeding or intestinal perforations [9] as major side effects when escalating the dose to the target. Gastroenteric toxicity after RT can represent a dramatic negative prognostic factor immediately or in case of new abdominal surgeries. In case of CIRT, these effects have been reported to be related to the smooth muscle layer radiation response with coagulation necrosis, arterial thrombosis, and atrophy of the intestinal epithelium [10]. The contiguity of radiosensitive organs to the CIRT tumor target in particular anatomical conditions might represent an important dose-limiting factor for CIRT. In both CIRT and conventional RT, anatomical spacer insertion has been reported to be a procedure to safely increase delivered dose to the tumor target and thus reach high doses to ensure adequate tumor control. Several methods or devices have already been reported to be used to locally displace the bowel loops or the rectum from the radiation field to reduce bowel and rectal complications and increase the total delivered radiation dose [11], especially for prostate cancer treatment with both conventional RT [12] and CIRT [13].

The aim of the current study is to analyze the feasibility of the insertion of a newly customized spacer prior CIRT in SC patients by laparotomic or hand-assisted laparoscopic approach, in terms of procedure validation and patient tolerance. Furthermore, the advantage in CIRT dose distribution due to our spacer silicon insertion is shown by comparing different treatment plans on CT scans from the same SC patient enrolled in the study, performed both before and after spacer insertion. This dosimetric advantage might translate in lower incidence of the digestive tract acute and late CIRT-related toxicity during patient treatment and subsequent follow-up.

## Methods

### Experimental study design and patient selection

Since 2014 to 2015, six consecutive patients were enrolled in the present study at San Matteo Hospital – Dept. of General Surgery among SC patients eligible for CIRT at CNAO. Patient characteristics and follow-up (up to 20 months) are presented in Table 1. This study is an experimental, exploratory, single-arm study approved by the Ethical Committee of the San Matteo Hospital and performed in accordance with the ethical standards of the Declaration of Helsinki. Specific written consent was obtained from all participants and data were anonymized. Exclusion criteria to enter the surgical study were principally related to chordomas anatomical position (below S3 was considered not eligible), technical contraindication to laparoscopic/open spacer positioning and patient general conditions allowing major surgery. Moreover, all the patients were considered eligible with a distance between the rectum and tumor inferior of 5 mm measured by preoperative CT scan and MRI. According to these exclusion characteristics, 14 patients have been ruled out and directly considered suitable for CIRT treatment. Patients who received previous abdominal surgery were selected for laparotomic approach due the high possibility of adhesions whereas patients that never underwent abdominal surgeries before were selected for hand-assisted laparoscopic surgery. After surgical spacer placement, SC patients were treated at CNAO within a phase II protocol for CIRT of bone and soft tissue sarcomas of the trunk approved by the institutional ethics committee of CNAO and authorized by the Ministry of Health. SC patients meeting all the following eligibility criteria were enrolled for CIRT at CNAO: no distant metastasis at the initial referral for treatment, no previous RT at the same site, a Karnofsky PS score >60, and a grossly measurable tumor. CIRT feasibility and optimal CIRT delivery are always discussed within CNAO clinical meeting within a board of radiation oncologists, radiologists, physicists, and surgeons. In case pre-enrollment patient CT or MRI shows chordoma located in close vicinity to the rectal wall or the bowel loop, the feasibility of spacer insertion and enrollment in the present collaborative study is routinely discussed.

**Table 1:** Patients characteristics

Variable	Patient (6)
Gender	
Male	2 pts (33.3 %)
Female	4 pts (66.6 %)
Age (median range)	45.3 years (20–55 years)
Follow-up	13.3 (6–18 months)
Previous abdominal surgery	
- Yes	3 pts (50 %)
Time of surgery—open approach (min)	133.33 (mean) – max 200/min 75
Post operative day (POD) of stay	7.66 (mean) – max 9/min 6
- No	3 pts (50 %)
Time of surgery—laparoscopic approach (min)	150 (mean) – max 240/min 70
Post operative day (POD) of stay	10 (mean) – max 12/min 9

### CIRT at CNAO

The specific technique of CIRT used at CNAO has been previously described in details [14]. For treatment plan preparation, CT and MRI data sets taken during the simulation phase were rigidly co-registered using the treatment planning system (TPS) (Syngo VC13, Siemens, Germany) to delineate the macroscopic tumor as the gross tumor volume (GTV), and the geometrical expansion of the GTV with its potential microscopic extension as the clinical target volume (CTV). A 4-mm expansion to the CTV was used to generate the planning target volume (PTV). The dose prescribed to the PTV (carbon physical dose in Gray × relative biologic effectiveness [RBE]) was 70.4 Gy (RBE) in 16 fractions of 4.4 Gy (RBE) each, 4 days a week on the basis of the results from the previous experience on CIRT trials of bone and soft tissue tumors adapted to CNAO clinical equipment [15]. RBE is variable depending on many factors incorporated into TPS (LEM I). Preclinical studies were performed in CNAO to assess the optimal dose prescription [16]. Normal structures were contoured for treatment planning (skin, intestine, rectum, sigma, bladder, cauda, and nerve roots), and dose constraints adopted as defined in previous clinical trials [7]. The CTV received at least 90 % of the prescribed dose. The modality of patient treatment and component system used for ensuring reproducibility of the patient setup position has been already described [17]. At the end of the CIRT course, the patients were closely monitored through physical examinations and MRI. Subsequent follow-up visits to check the progress of SC patients were performed in CNAO at least every 3 months. CIRT toxicity during treatment and at each follow-up is routinely scored by means of the Common Terminology Criteria for Adverse Events (CTCAE) v4.0 scale.

### Silicon spacer preclinical assessment for radiation hardness and stability

Prior to the clinical use of the spacer, several measurements were performed to evaluate its physical stability during and after CIRT, as well as its main properties potentially affecting clinical protocols for CIRT when exposed to particle beams. Firstly, high-energy scanned carbon ion beams (208.6 MeV/u) produced by the CNAO synchrotron were used to expose a spacer sample, consisting of five overlapping sheets of material (1.1-mm thick each, 10 × 5 cm^2^ area, closely simulating the spacer intended to use in the clinical setup) to a single dose of 135 Gy, much exceeding the therapeutic dose level, on a 3 × 3 cm^2^ portion of the spacer. Then, measurements of the particle range in the spacer material were performed at the carbon ion beam energy of 280 MeV/u using the Peakfinder dedicated device (PTW, Freiburg, Germany), in terms of water equivalent path length (WEPL) [18]. Finally, the spacer underwent computed tomography (CT) scan, acquired using the head protocol adopted for planning CT scans in the clinical routine at CNAO (120 kVp, 320 mAS, 2 mm thickness, H31s reconstruction filter, 500 mm field of view).

### Silicon sheet spacer modeling

**Fig. 1:**
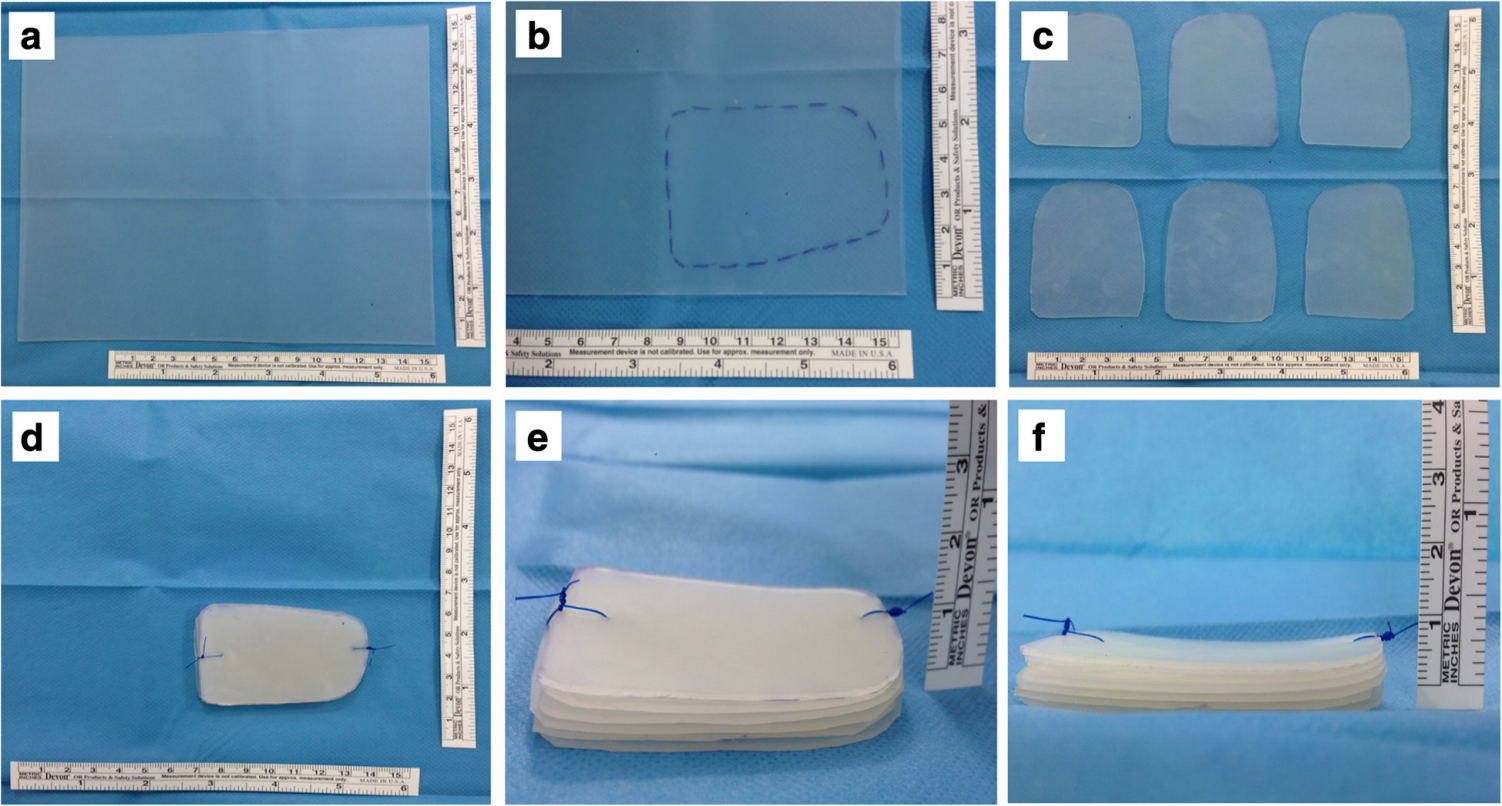
Shaping and preparation of patient-tailored silicone spacer. Starting from a single, 1-mm width and 10 × 10 cm silicone sheet (**a**), the first plate has been created and shaped directly from tumor features (**b**) with surgical blade. Subsequently, the optimal width for the final spacer has been chosen (usually around 5 mm) and then singles pre-modeled silicone sheets (**c**) are stacked up and fixed by 1-0 Prolene suture (**d–f**)

For each patient, silicone spacer was shaped according to pre- and intraoperative findings from a 10 × 10 cm silicone sheet with a width of 1 mm (Distrex, Padua, Italy). From one sheet, it was possible to obtain up to six surgical spacers, for a total of 6-mm thickness. Contoured spacers were then simply fixed all together by Prolene suture (Fig. 1). This procedure allowed the surgeon to decide the desired final width of the spacer based on patient anatomical features. For the spacer design, we decided to use silicone rubber as material for its biocompatibility, temperature, and chemical resistance other than mechanical properties. Biocompatibility, defined as “the ability of a material to perform its function with an appropriate host response in a specific situation” [19], perfectly mirrors the main quality of this material with a nonirritating and nonsensitizing behavior inside the body, after its placement. Mechanical quality is an additional important feature of this polymer ensuring a high tear and tensile strength, up to 250 ppi and 1500 psi, respectively [20]. Silicone’s intrinsic features, such as tenderness and flexibility, have been considered adequate for the appropriate accommodation of the spacer on the tumor bulge.

### Surgical procedure

For the surgical procedure, all the patients underwent general anesthesia with tracheal intubation, indwelling bladder catheter and were placed in a decubitus dorsal position with their arms next to their body. At the time anesthesia was induced, intravenous antibiotic prophylaxis with first-generation cephalosporin was established.

For laparotomic procedures, an umbilical/pubic midline laparotomy was performed with direct access to the abdominal cavity.

**Fig. 2:**
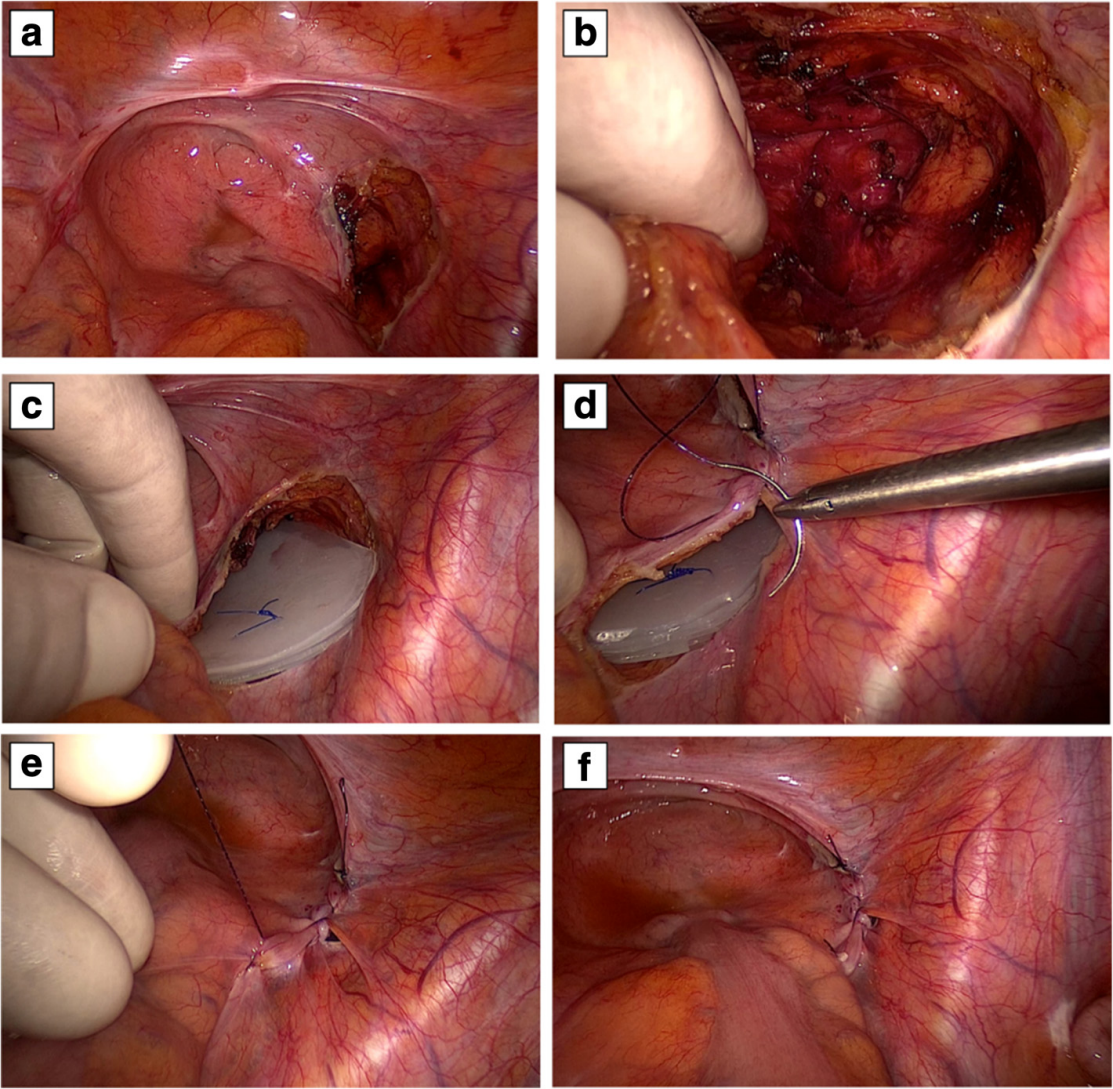
Laparoscopic hand-assisted silicone spacer placement. The peritoneum has been dissected directly above of the tumor area intended to dress through the spacer (**a**, **b**). The molded spacer has been located inside the created “peritoneal pouch” and then fastened by continue 3-0 Prolene suture (**c**–**e**). The conclusion of peritoneal closure ensures the optimal fixation and stability of the silicone spacer (**f**)

For laparoscopic procedures, trocars and hand-assisted device were placed as follows. Two 10 mm optical trocars were placed in the subumbilical region and in the right lower quadrant (right iliac fossa), respectively. The hand-assisted device was placed at the level of the left rectus border (Fig. 2). Insufflation into the abdominal cavity with carbon gas, reaching a maximum abdominal pressure of 15 mmHg was performed. After the pneumoperitoneum was achieved, a 10 mm 30° optic was introduced inside the abdominal cavity through the umbilical trocar.

Trendelenburg position was achieved in order to dislocate intestinal loops for both.

In both surgical setup (open or laparoscopic), a retro-rectal pouch was created by blunt dissection of parietal peritoneum and the spacer was then inserted and fixed with Ethilon 3/0 just rebuilding the peritoneum pouch.

## Results

### Silicon spacer preclinical assessment for radiation hardness and stability

Visual inspection after the irradiation and 1 month later did not reveal any macroscopical differences between the spacer at the level of exposed and unexposed areas, in terms of sample transparency and color, nor any variation to the tactile or heating effect. The same WEPL value, equal to 1.084, was measured within both previously exposed and unexposed portions of the spacer. Finally, the difference between the mean Hounsfield units (HU) calculated within the irradiated and non-irradiated areas fell well within the statistical noise (418 ± 7 as one standard deviation versus 416 ± 8), thus showing no radiation-induced variation in the x-ray attenuation properties (i.e., electron density) of the investigated material.

### Patient workout and surgical procedure

**Fig. 3:**
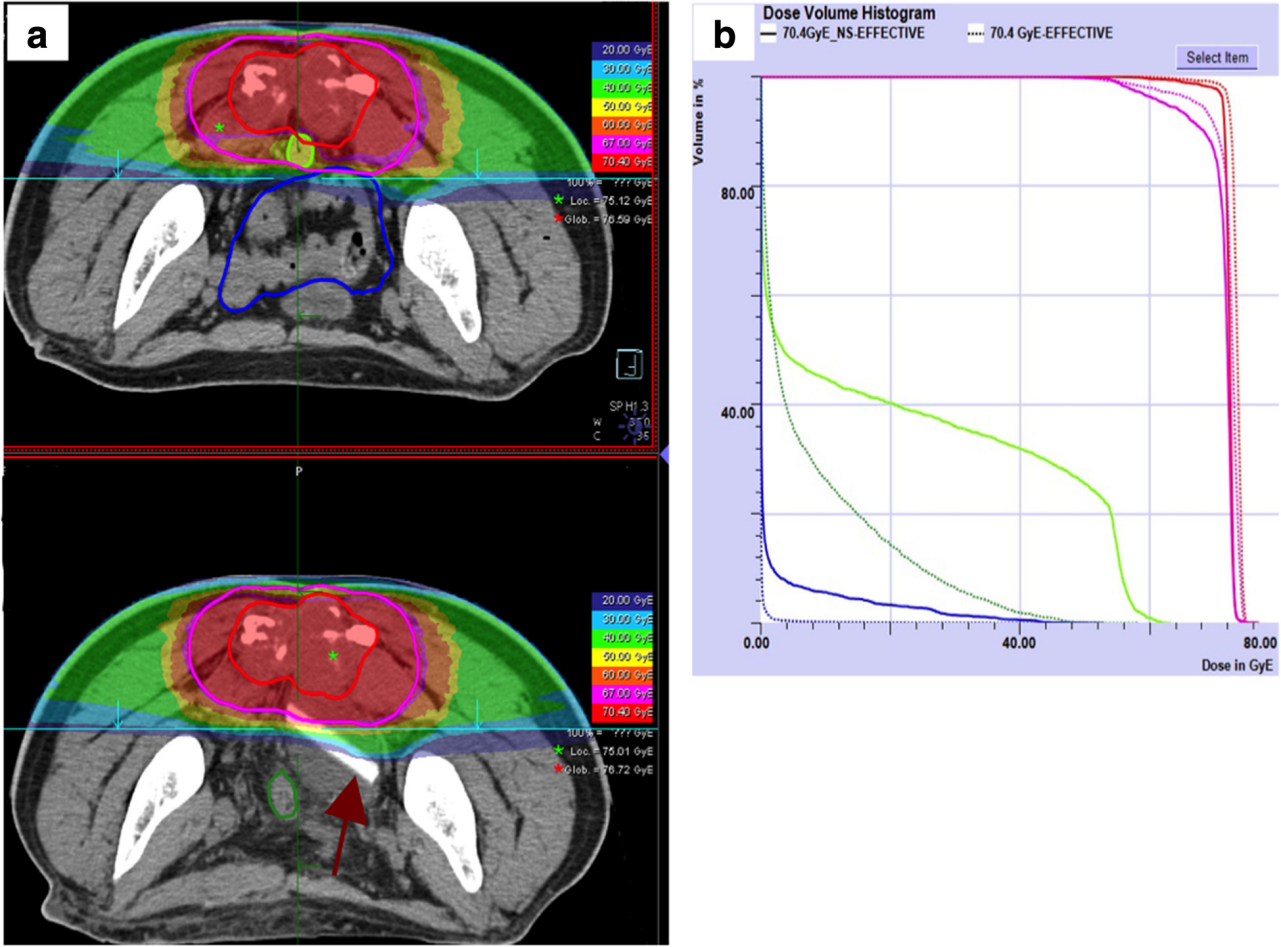
Plan comparison study on different CT from the same patient selected for spacer positioning at CNAO. **a** Different CIRT plans depicted on axial images at the same anatomical level for the same chordoma patient. *Red lines* for GTV and *green lines* for CTV are included within the prescription dose isodose in both CIRT plans of 70.4 Gy (RBE). In the *upper panel*, the digestive area (rectal wall in *green* and intestine in *blue*) is contiguous to the prescribed dose isodose. In the *lower panel*, the digestive tract is far away from irradiated area due to the spacer (pointed by a *red arrow*). **b** Comparison of DVH for GTV (*red*), PTV (*pink*), and digestive tract (rectum *green*, intestine *blue*) dose coverage from the two CIRT plans compared in **a**. Coverage of the tumor is higher with the spacer CIRT plan (*dotted lines*) compared to the plan without it (*continuous lines*). With the CIRT spacer, plan dose sparing to the digestive tract is achieved

According to eligible criteria, a series of six patients were enrolled in this study. Three patients underwent open surgical spacer insertion whereas the other three hand-assisted laparoscopic positioning. All the patients underwent silicon spacer placement without any intraoperative complications. Laparotomic and laparoscopic spacer placement showed different advantages principally secondary to patient characteristics. Laparotomic surgery has been used for patients who underwent previous abdominal surgeries in order to by-pass adhesions presence. For patients who face abdominal surgery for the first time, laparoscopic approach was preferred. Compared to laparotomic approach, hand-assisted laparoscopy allowed surgeons direct hand contact with the operative field, maximizing tactile feedback and minimizing surgical injury. Silicone spacer placement demonstrated to be safe for the patients during treatment and for the entire follow-up period of time. Mean postoperative days (mPOD) before CIRT were 7.66 ± 1.24 for the patients who underwent open approach and 10 ± 1.41 for the others undertaking the laparoscopic method. For the present study, all patients CT and MRI during and after CIRT were reviewed in order to assess any prosthesis displacement or any other anatomic changes. During CIRT, the presence of the spacer keeps digestive tract far away from the irradiated area; thus, the radiation field is unaffected by rectum filling or intestine movement (Fig. 3a). Patient imaging did not show anatomic variations, and silicone spacer placement has been demonstrated to be safe for the patients for the entire follow-up period of time. Patient enrolled in the study did not show any gastrointestinal toxicity during CIRT or at follow-up, such as gastrointestinal bleeding, fecal urgency, or lower abdominal cramping (median follow-up time 13.3 months, range 6–18 months). Any spacer dislocation has been observed during the follow-up period.

### Laparotomic representative case

Under general anesthesia, an umbilical/pubic laparotomy was performed with direct access to the abdominal cavity that was then manually explored with SC identification. Trendelenburg position was then achieved. After identification of the iliac vessels, the ureter and the gonadic vessels, the peritoneum was bluntly dissected at the tumor level. The ureter was gently isolated, and the peritoneum was detached from the tumor to create the appropriate zone for the spacer positioning. Up to six silicon sheets (1 mm of width each) were shaped to perfectly fit the created area between the parietal peritoneum and chordoma surface. Silicon spacer was then located, and the peritoneum was closed on it by continue Vicryl suture (Vicryl 3/0). No other surgical procedures were needed in order to secure the device. The abdomen was then closed by anatomical layers.

### Laparoscopic representative case

Under general anesthesia, the patient was fixed to the table and laparoscopic trocars were positioned as follows: the first 12-mm port (Fig. 2a) for the laparoscope was placed at the umbilical site, the second (Fig. 2b) 10-mm was placed at the right side of the rectus muscle, and finally, the GelPort device, after a 6.5-cm length skin incision, was sited at the left side of the rectal muscle (Fig. 2c). The iliac vessels, the ureter, and the gonadic vessels were identified, and the peritoneum covering the tumor was dissected preparing a “peritoneal pouch”. Finally, the silicon-based spacer was customized to fit on it. Silicon spacer was then located, and the peritoneum was closed on it by continue Vicryl suture. Laparoscopic and GelPort accesses were then closed.

### CIRT plans comparison

In order to show the effect of the spacer insertion on CIRT planning, we selected one of the six patients enrolled in our study to perform the same CIRT plan recalculation on a CT from the same patient but undertaken before the silicon spacer insertion. In Fig. 3a, representative CT slices from the two CIRT plans show how the spacer insertion allows separation of the rectal wall from the irradiated area, with increasing of the tumor coverage with the maximum prescribed curative dose. Furthermore, a comparison of dose volume histograms (DVH) from the two plans (Fig. 3b) shows little effect on the tumor coverage but great sparing of the rectum and intestine in case of spacer insertion.

## Discussion

Beyond histological confirmation, surgical treatment is still quoted as the standard cure management for SC [21]. Surgical radical resection offers a more extended disease-free period compared to R1 resection, but often this treatment cannot be achieved due to the chordoma volume at the time of diagnosis [22] and to the possible involvement of the upper levels of the sacrum or the lumbar spine [23]. As of now, sacrectomy with R0 resection margins offers the best long-term prognosis [24] even with its aggressiveness and the deriving low quality of life with an overall 5 and 10-year survival rates of 45–77 and 28–50 %, respectively [25]. Most important limitative surgical outcomes are urinary and bowel incontinence [21], wound infections, chronic neuropatic pain, and paresis.

The consensus meeting of 2013 on chordomas organized by the European Society for Medical Oncology (ESMO) established that surgery and high-dose RT are the treatment mainstays for localized chordomas, both of which have curative potentials [26]. For SC patients, CIRT represents an innovative and useful weapon, which is worthy to be further investigated [27] within controlled clinical studies. SC is characterized by very poor radiosensitivity to “standard” photon RT, but carbon ion and protons RT allow delivering high conformal dose to the tumor due to their intrinsic physical properties increasing the curative potential of the RT treatment course. Moreover, as deeply reviewed by Durante M., carbon ion beams (similar to the other members of the heavy ions family) deliver a high-conformed energy into the treatment area combined with a greater radiobiological effectiveness [28], thus increasing the tumoricidal effect probability. Main injuries to the small and large intestines due to RT are related to fibrosis and vascular insufficiency resulting in a status of chronic ischemia [29]. This condition can then lead to chief problems such as intestinal obstruction, severe bleeding, or fistula formation all the length way of the intestine even if with distinctions between the terminal ileum, cecum, sigmoid, and rectum [30]. Undoubtedly, the greatest concern regarding the application of CIRT for the treatment of tumor target localized in the abdomen remains gastro-urinary tract (GUT) toxicity. In particular, the proximity of SC to the sigma-rectum tract forces the radiation oncologist to reduce the tumor dose coverage in the tumor area closed to the GUT critical organs, limiting the curative potential of the delivered treatment. With our study and CIRT plans comparison, we have shown that our silicon spacer insertion might help in delivering high curative doses to the SC sparing the GUT critical organs. Evaluations and instrumental measurements performed on the spacer, prior its implantation, showed that the spacer is not affected by radiation damage when exposed even to high doses of ion beams and therefore can be safely used in the clinical practice to help sparing healthy tissues from unwanted high doses during CIRT.

From urological and gynecologic experience deriving from RT for several tumoral diseases, different approaches have been used to displace the intestine outside the irradiation-targeted area [31]. We believe that the design and positioning of a spacer represents the solution deliver safe CIRT for SC patients, and, in particular our spacer might be a valuable tool to separate the RT-targeted tumor volume from the bowel loop. We also consider our findings very helpful not only for CIRT but also for conventional 3D-conformal RT where high radiation doses are delivered with curative intent to tumor targets in the abdomen. To our knowledge of the literature, the number of cases presented in the present work is the largest ever published by a single center using silicon spacers, and represents the first report using the minimally invasive technique (in three out of six cases) for silicon spacer placement prior CIRT for SC. Mima et al. described 17 cases of laparotomic polytetrafluoroethylene (PTFE) spacer placement with good results [22]. We considered to use silicon instead of PTFE for three reasons: (a) silicon is a biocompatible material worldwide used for surgical implants; (b) the cost effectiveness of silicon is superior to PTFE (the commercial price of silicon a sheet is 6/10 times less compare to PTFE); and (c) we found silicon superior to PTFE in terms of modeling and customization to the anatomy of the patient. Analyzing our experience, we can state that the use of a silicon spacer adapted to the anatomical features of the SC is safe and feasible, without any complications neither intra nor postoperative. The hand-assisted minimally invasive technique has been feasible and safe by ensuring correct positioning of the spacer, and we chose the open technique in cases of previous abdominal surgery because of possible peritoneal adhesions. Hand-assisted laparoscopy provided optimal tissue palpation and retraction, tactile feedback, 3-D spatial orientation, and blunt dissection of the involved anatomical structures. The use of the non-dominant hand directly into the operative field played a pivotal role for the choice of this unique minimally invasive approach that combined the finest aspects of the “classical” open surgery with those of conventional laparoscopy.

## Conclusions

Our preliminary clinical study shows how surgical spacer placement before CIRT could be a feasible approach to achieve a patient-tailored treatment minimizing RT-related toxicity. Shaping of the spacer, directly modeled on the anatomical feature of the patient, allows to produce patient-specific tailored device. The overall safety of the spacer seemed to be excellent with a 100 % successful rate whereas no adverse effects were noticed in any patient treated. Spacer positioning increases the distance between chordoma and the most critical organs at risk reducing at the same time their exposure to CIRT and, therefore, the probability of any hazardous toxicity during and after therapy.

## Abbreviations

CIRT, carbon ion radiotherapy; CNAO, National Center for Oncological Hadrontherapy; CTCAE, Common Terminology Criteria for Adverse Events; CTV, clinical tumor volume; GTV, gross tumor volume; PTV, planning target volume; RBE, relative biologic effectiveness; RT, radiotherapy; SC, sacral chordoma; TPS, treatment planning system; WEPL, water equivalent path length
