# Erratum to: Surgical spacer placement prior carbon ion radiotherapy (CIRT): an effective feasible strategy to improve the treatment for sacral chordoma

**DOI:** 10.1186/s12957-016-1020-4

**Published:** 2016-10-12

**Authors:** Lorenzo Cobianchi, Andrea Peloso, Barbara Vischioni, Denis Panizza, Maria Rosaria Fiore, Piero Fossati, Viviana Vitolo, Alberto Iannalfi, Mario Ciocca, Silvia Brugnatelli, Tommaso Dominioni, Dario Bugada, Marcello Maestri, Mario Alessiani, Francesca Valvo, Roberto Orecchia, Paolo Dionigi

**Affiliations:** 1 Department of Clinical, Surgical, Diagnostic and Paediatric Sciences, University of Pavia, Pavia, Italy; 2 IRCCS Policlinico San Matteo Foundation, General Surgery 1, Pavia, Italy; 3 Department of Radiation Oncology and Medical Physics, Centro Nazionale Adroterapia Oncologica (CNAO), Pavia, Italy; 4 Department of Radiation Oncology, European Institute of Oncology (IEO), Milan, Italy; 5 IRCCS Policlinico San Matteo Foundation, Department of Onco-Hematology, Oncology Section, Pavia, Italy; 6 Department of Surgical Science, University of Parma, Parma, Italy; 7 Department of General Surgery, IRCCS San Matteo Foundation, University of Pavia, Piazzale Golgi, Pavia, 27100 Italy

## Erratum

Following publication of the original article [1] it was brought to our attention that there had been some confusion surrounding the presentation of the author names in the author list, leading to the first name and family name of each author being misplaced. Please see below for the correct presentation of the author names as they should appear in the author list:

Lorenzo Cobianchi^1,2,7*^, Andrea Peloso^1,2^, Barbara Vischioni^3^, Denis Panizza^3^, Maria Rosaria Fiore^3^, Piero Fossati^4^, Viviana Vitolo^3^, Alberto Iannalfi^3^, Mario Ciocca^3^, Silvia Brugnatelli^5^, Tommaso Dominioni^1,2^, Dario Bugada^6^, Marcello Maestri^1,2^, Mario Alessiani^1,2^, Francesca Valvo^3^, Roberto Orecchia^3,4^ and Paolo Dionigi^1,2^

This has now been updated on the BioMed Central website.
